# Amplifying Lamb Wave Detection for Fiber Bragg Grating with a Phononic Crystal GRIN Lens Waveguide

**DOI:** 10.3390/s22218426

**Published:** 2022-11-02

**Authors:** Chia-Fu Wang, Junghyun Wee, Kara Peters

**Affiliations:** Department of Mechanical and Aerospace Engineering, North Carolina State University, Raleigh, NC 27695, USA

**Keywords:** structural health monitoring, guided wave imaging, phononic metamaterials, fiber Bragg grating sensors

## Abstract

This paper demonstrates that a graded-index (GRIN) phononic lens, combined with a channel waveguide, can focus anti-symmetric Lamb waves for extraction by a detector with strong directional sensitivity. Guided ultrasonic wave inspection is commonly applied for structural health monitoring applications; however, obtaining sufficient signal amplitude is a challenge. In addition, fiber Bragg grating (FBG) sensors have strong directional sensitivity. We fabricate the GRIN structure, followed by a channel waveguide starting at the focal point, using a commercial 3D printer and mount it on a thin aluminum plate. We characterize the focusing of the A_0_ mode Lamb wave in the plate, traveling across the GRIN lens using 3D laser Doppler vibrometry. We also measure the extraction of focused energy using an FBG sensor, examining the optimal sensor bond location and bond length in the channel of the waveguide for maximum signal extraction. The measured amplification of the ultrasound signal is compared to theoretical predictions. The results demonstrate that significant amplification of the waveform is achieved and that selecting the location of the FBG sensor in the channel is critical to optimizing the amplification.

## 1. Introduction

Guided Lamb wave imaging is commonly used for structural health monitoring (SHM) of large structures since Lamb waves can propagate large distances in thin structures [1,2,3]. Lamb waves can be actively generated in the structure using small actuators, and their propagation is altered due to the presence of damage in a structure. One common example of a detector is an optical fiber Bragg grating (FBG) sensor [4]. One benefit of using FBG sensors is that multiple sensors can be multiplexed along a single fiber so that many sensing locations can be provided with only one lead-in and -out connection [5]. In addition, FBG sensors have strong directional sensitivity with respect to ultrasonic wave detection; therefore, they can filter out signals not directly from the actuator, for example, unwanted reflections from boundaries [5]. Similarly, directional sensors can be arranged in a rosette or phased array configuration to identify the location of impact or acoustic emission sources [6,7,8].

The peak amplitude of Lamb waves excited by a point source decays quickly with the distance from the source due to the spreading of the wave and material attenuation [9,10]. Therefore, extracting relevant information from the Lamb wave at a significant distance away from the source often requires amplification of the signal. The cylindrical geometry of optical fibers also reduces signal transmission from the structure to the fiber due to wave coupling through the adhesive, compared to a planar device (such as a PZT). Therefore, amplification of the Lamb wave prior to capture by the FBG sensor is critical. Our research group investigated the remote bonding of an FBG sensor to increase the FBG signal-to-noise ratio in guided wave detection for structural health monitoring applications [11,12]. In this configuration, the ultrasonic Lamb waves were first converted into propagating longitudinal ultrasonic modes in the optical fiber. The signal was then detected by an FBG sensor further along the optical fiber. The resulting FBG sensitivity to the ultrasonic signal was higher than for conventional surface bonding of the FBG. The remote bonding concept was also coupled with a planar ultrasonic horn to amplify the ultrasound signal prior to coupling into the optical fiber [13]. Amplification of the sensor output was achieved; however, many spurious waveforms were introduced due to reflections off the boundary of the ultrasonic horn, which sometimes interfered with the signal to be detected.

Previous researchers demonstrated that a graded-index (GRIN) lens that manipulates Lamb waves could be formed by a phononic crystal lattice of pillars or holes on the surface of a structure [14,15,16,17]. The phononic crystal unit cells change the local propagation velocity of anti-symmetric Lamb waves as a function of the pillar or hole dimensions and material [14]. The local effective refractive index of a unit cell is defined as the ratio of the Lamb wave phase velocity in the homogenous host structure and in the unit cell comprised of the phononic crystal and host structure [14,16,18]. In a phononic crystal GRIN lens, the local effective refractive index of each unit cell is gradually varied to achieve a specific effective refractive index profile. For pillar systems, this change in the effective index can also be enhanced by choosing pillar dimensions such that the longitudinal resonance of the pillar is excited at the Lamb wave frequency [19]. Similar to photonic systems, the hyperbolic secant profile of the effective refractive index is most commonly applied because it focuses the propagating Lamb waves with minimal transverse aberration and focal spot size [20].

**Figure 1:**
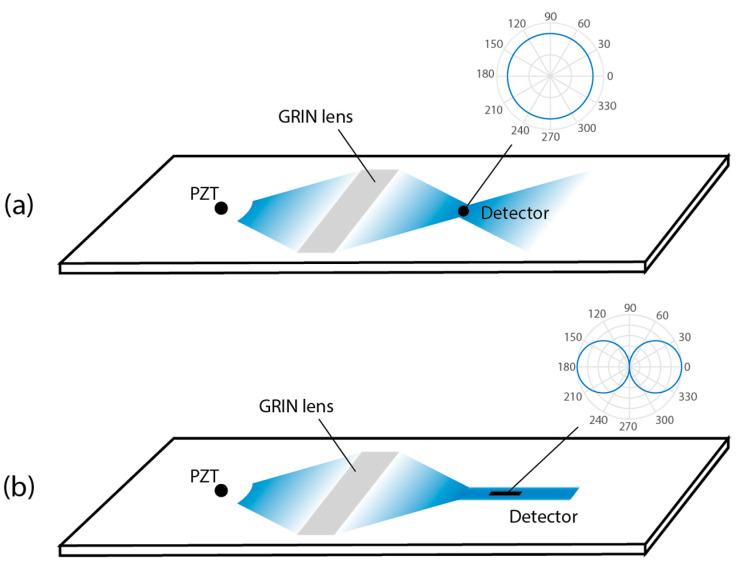
(**a**) Omni-directional ultrasound detector in GRIN lens focal spot. Inset shows the sensitivity of the detector. (**b**) Directional ultrasound detector in guided wave region after GRIN lens focal spot.

Assuming the actuator and detector are far apart, the input Lamb wave is essentially a plane wave prior to interacting with the phononic crystal. If the detector is small relative to the focal spot size and has omnidirectional receiving characteristics, the detector is best placed at the focal point of the GRIN, as shown in Figure 1a. In this case, the detector essentially acts as a point sensor, and the detected signal is amplified with minimal distortion. These assumptions are reasonable for many detectors, such as small PZT discs or external laser Doppler vibrometry. However, when the detector has a strong directional sensitivity or is larger than the focal spot size, the benefits of the focusing behavior may be outweighed by the resulting distortion to the detected signal. For example, the length of the FBG sensor is on the order of a centimeter; therefore, the diverging Lamb wave would create a non-uniform wave amplitude along the FBG [5]. This effect is increased by the fact that the FBG sensitivity drops quickly with input angle, so the collected signal would decrease along the FBG length as well. For this case, a better strategy is to collimate the focused Lamb wave into a guided planar wave after the focal point and to place the detector in the collimated region, as shown in Figure 1b.

In this paper, we apply the concept of Figure 1b to focus A_0_ Lamb waves in a plate using a 3D printed GRIN lens and waveguiding channel phononic crystal structure prior to extraction with an optical fiber and detection with an FBG sensor. In order to accommodate the strong directional sensitivity and linear geometry of the FBG sensor, we confine the focused wave into a waveguiding channel after the GRIN focal point, then surface bond a segment of the optical fiber in the channel. We experimentally confirm the focusing performance of the GRIN lens on the A_0_ mode at the operational frequency by 3D laser Doppler vibrometry (LDV). We then add a 3D-printed waveguiding channel after the focal spot and demonstrate the waveguiding performance of the channel. Finally, we measure the amplified FBG response in the waveguide channel and compare it with theoretical predictions.

## 2. Lamb Wave Focusing with GRIN Lens

**Figure 2:**
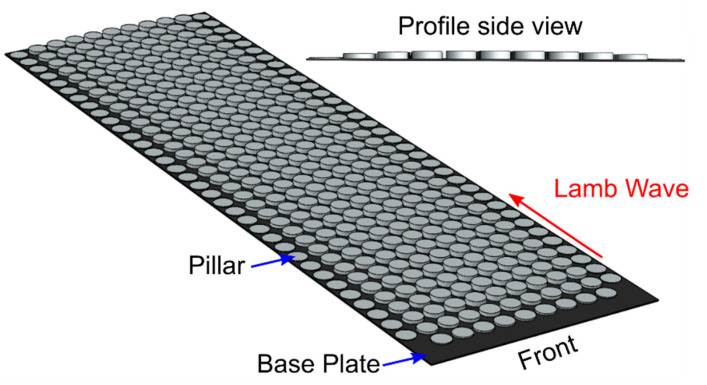
GRIN structure with tapered pillar height on the front edge. Pillar height profile for one row is shown.

The 3D-printed GRIN structure with 36 unit cells in the propagation (x) direction and 11 unit cells in the perpendicular (y) direction is shown in Figure 2 and was modeled from the example of Tol et al. [14]. The unit cells have a lattice constant (unit cell dimension) of 5 mm and a pillar diameter of 4.375 mm. This lattice constant was chosen from the dispersion curve for the plate. The first bandgap in the dispersion curve starts at 55 kHz; therefore, the theoretical operational frequency for the unit cell is below 55 kHz. The length and width of the GRIN structure are 180 and 55 mm. The designed GRIN structure was printed from acrylonitrile butadiene styrene (ABS) material using a 3D printer. The 3D printer prints in 0.127 mm layers and extrudes ABS at a raster width of 0.254 mm. A base plate of 0.254 mm thickness was printed below the pillars to support the 3D printing of the lens and bonding it to the aluminum plate. This plate thickness was the minimum value that could be printed with consistent thickness over the area of the lens.

The height of the pillars was chosen to achieve a hyperbolic secant distribution in the local refractive index for the A_0_ mode across the width of the lens (in the y direction),

n(y) = n_0_ sech(αy), (1)

where *n_0_* is the local refractive index along the center axis, and *α* is the gradient coefficient [14]. The pillar heights for all columns are listed in Table 1. The first few rows of the GRIN lens were also tapered in pillar height to minimize reflections from the front edge of the lens due to the impedance mismatch between the plate and the combined plate–GRIN lens system. The height of the pillars was linearly increased from 0 mm (only the base plate present) to the full height of the pillars in column 4.

**Figure 3:**
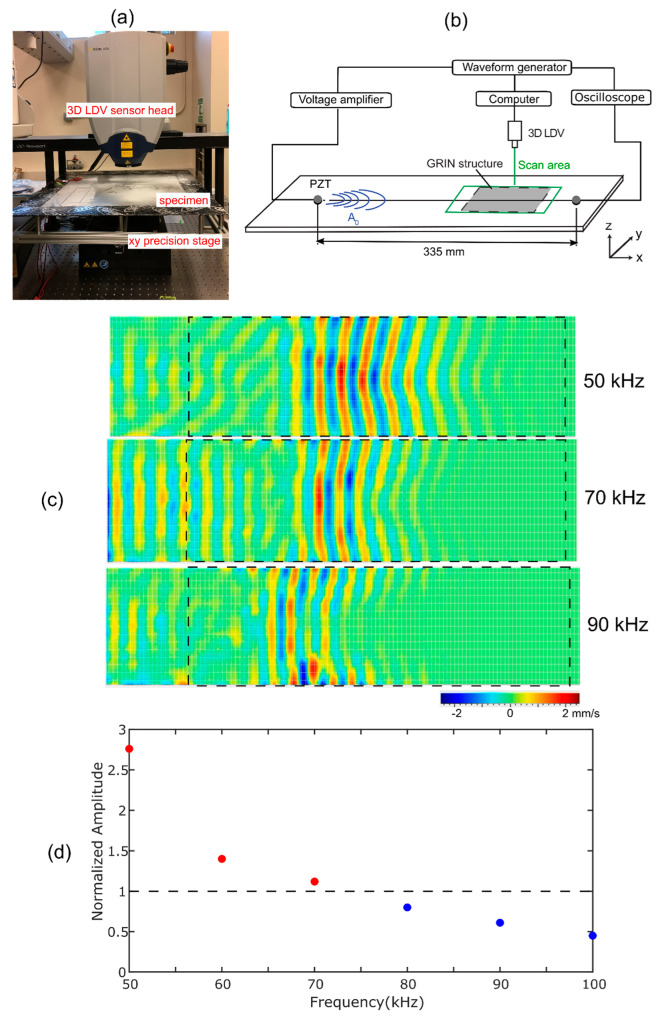
Measurement of the focus performance of the GRIN structure for A_0_ mode: (**a**) photograph of the 3D LDV measurement system. (**b**) Schematic of experimental setup. (**c**) LDV measurements of *z*-directional surface velocity due to propagating A_0_ Lamb waves. Wave propagation is from left to right. Dashed rectangle is the GRIN region. 50 kHz wave recorded at 275 µs; 70 kHz wave at 216 µs; 90 kHz wave at 183 µs. (**d**) Amplitudes of the output PZT response as a function of the input A_0_ mode frequency, normalized to reference case without GRIN structure. Red data points have an amplitude above and blue data points below the reference value respectively.

Figure 3a,b shows the experimental setup to measure the focus performance of the GRIN structure on the A_0_ Lamb wave propagating through the 6061 aluminum plate with 0.8 mm thickness. The dimensions of the plate are 609.6 mm × 609.6 mm. An elastomeric damping material (Dynamat^®^) wasused to cover the boundary of the aluminum plate on both top and bottom surfaces in order to reduce back-reflections. Two PZT actuators were bonded on each side of the aluminum plate at the same location and actuated out-of-phase in order to excite A_0_ mode Lamb waves. The PZT actuators were excited with a 5.5 cycle Hanning windowed burst sine wave for all experiments. The excitation signal was input to the PZTs from an arbitrary waveform generator (AWG) via a voltage amplifier.

The A_0_ mode propagation through the aluminum plate was measured using the 3D laser Doppler vibrometry (LDV) (MSA-100-3D, Polytec GmbH, Waldbronn, Germany) system shown in Figure 3a. LDV scanning was performed on the opposite side of the plate from the GRIN lens. The aluminum plate was placed under the 3D LDV sensor head on the XY precision stage. The *x*-direction was aligned with the propagation direction, the *y*-direction in the plane of the plate was perpendicular to the propagation direction, and the *z*-direction was perpendicular to the surface of the plate. The input excitation signal from the waveform generator was time-synchronized with the output measurement of the 3D LDV. The 3D LDV scanning region is shown as the green box in Figure 3b. The scan area was spray-coated with a thin layer of white powder (Weld Check^®^ Developer, CRC) in order to create uniform light scattering from the surface of the specimen because the aluminum plate is excessively reflective. The distance between each scanning point was 2 mm along the wave propagation direction and 3.5 mm along the direction perpendicular to the wave propagation.

To bond the GRIN structure to the plate, Kapton^®^ tape was first used to delineate the GRIN lens region on the plate. Epoxy resin (West System^®^ 105-205, West Marine, Fort Lauderdale, FL, USA) was spread on the rectangular area and scraped with a metal plate to produce a controlled thickness, 0.06 mm, i.e., the thickness of the Kapton tape. Then, the Kapton tape was removed, and the GRIN structure was placed on the epoxy. A constant weight was applied from the top of the GRIN structure to provide consistent pressure. The epoxy was allowed to cure for 24 h. The distance from the PZT actuators to the leading edge of the GRIN structure was 150 mm.

Figure 3c plots the measured out-of-plane velocities in the scan area at three different PZT excitation frequencies. The selected times for the images were different between the frequency cases because their propagation speeds were different. The 50 kHz A_0_ mode entered the GRIN lens, and its wavefront gradually bent due to the GRIN structure. However, when the input wave frequency was close to the bandgap lower frequency, 70 kHz, the A_0_ mode was attenuated as it propagated through the GRIN structure, and a significant amount of the energy was reflected from the front edge of the GRIN structure due to the bandgap effect. Above the bandgap, 90 kHz, the mode entered the GRIN lens but was highly attenuated as it propagated through the GRIN structure. These observations are consistent with the expected behaviors from the simulations of Tol et al. [14]. Based on these results, we chose 50 kHz as the A_0_ mode excitation frequency for later experiments.

To compare the A_0_ mode amplitude at a single point with and without the GRIN lens, the A_0_ mode was also measured using a PZT sensor at 335 mm away from the PZT actuator, as shown in Figure 3b. The oscilloscope acquisition was time-synchronized with the PZT excitation of the AWG. Figure 3d plots the peak-to-peak amplitudes of the signals measured with the PZT sensor with varying input A_0_ mode frequency. The values were normalized to the peak-to-peak amplitude of the PZT measurement without the GRIN structure present. This normalization accounted for the changing output of the actuator PZT and sensitivity of the measurement PZT with frequency. We observed that the amplitude of the A_0_ mode after the GRIN lens was higher than the reference case for 50 and 60 kHz and decreased with increasing frequency. This behavior was a combination of the GRIN lens focusing on the A_0_ mode, which was strongest at 50 kHz, and the increasing attenuation of the mode with the higher frequency seen in Figure 3c. Above 70 kHz, the measured amplitude was actually lower than for the reference case.

**Figure 4:**
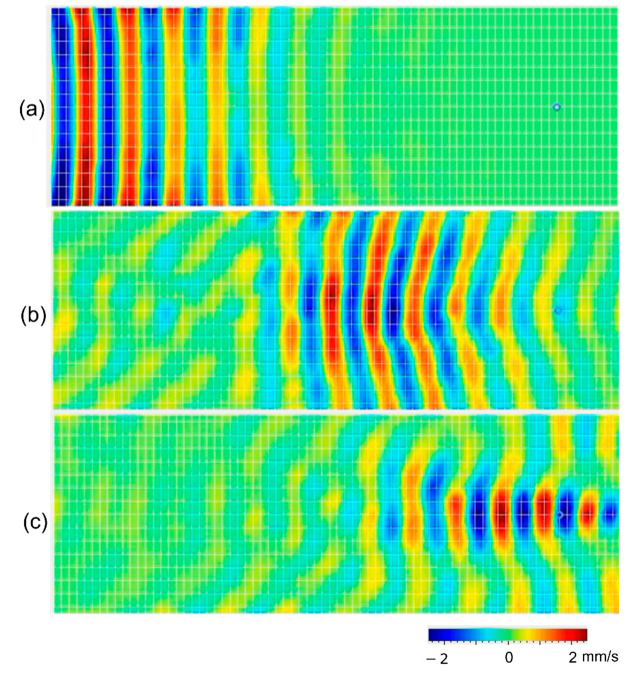
LDV measurement showing the z-directional surface velocity of 50 kHz A_0_ mode Lamb waves at (**a**) 210 µs, (**b**) 284 µs, and (**c**) 323 µs.

Finally, we characterized the focusing of the wave by the GRIN structure at 50 kHz. Figure 4 plots the measured z-directional surface velocity from the LDV. Figure 4a plots the wave velocity at 210 µs when the A_0_ Lamb wave in the plate started to enter the GRIN structure. The Lamb wave approximately entered the GRIN structure as a plane wave. As the wave propagated into the structure, Figure 4b,c shows the wavefront gradually bending due to the refractive index profile. Finally, the waves were most focused at approximately 130 mm into the GRIN structure, as indicated with a blue circle, where the maximum amplitude of z-direction surface velocity was measured.

**Figure 5:**
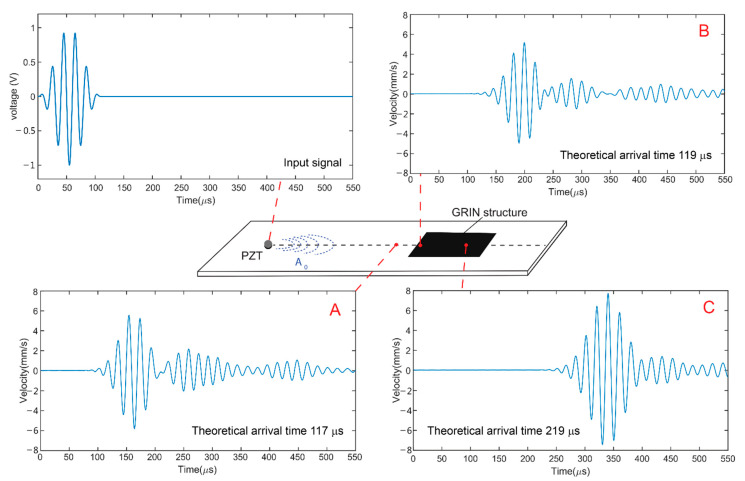
Input signal to the PZT actuator and LDV measurements of the z-direction velocity at three locations on the plate.

We also performed single-point LDV measurements at three different locations on the plate, as shown in Figure 5, to compare the A_0_ waveforms. Point A is in front of the leading edge of the GRIN structure, point B is immediately after the leading edge, and point C is at the focal point. Figure 5 plots the z-directional surface velocity at those three locations as well as the input signal to the PZT actuator. Based on the group velocity of the A_0_ mode from the dispersion curve, the theoretical arrival times of the A_0_ mode packet at points A, B, and C were 117, 119, and 219 μs, respectively. Although the PZT actuator was excited with a 5.5 cycle Hanning windowed burst sine wave, the LDV continued to measure some vibrations after the original wave packet. This could be due to the ringing of the PZT actuator because the same vibrations were collected from all three measurement locations. The peak-to-peak amplitudes of the A_0_ mode packets were 96, 121, and 242 mm/s for points A, B, and C, respectively. These measurements show that the A_0_ mode amplitude of point C was increased by 35% compared to that of point A and by 50% compared to that of point B; therefore, the A_0_ Lamb wave was focused by the GRIN structure.

## 3. GRIN Structure with Waveguide Channel

**Figure 6:**
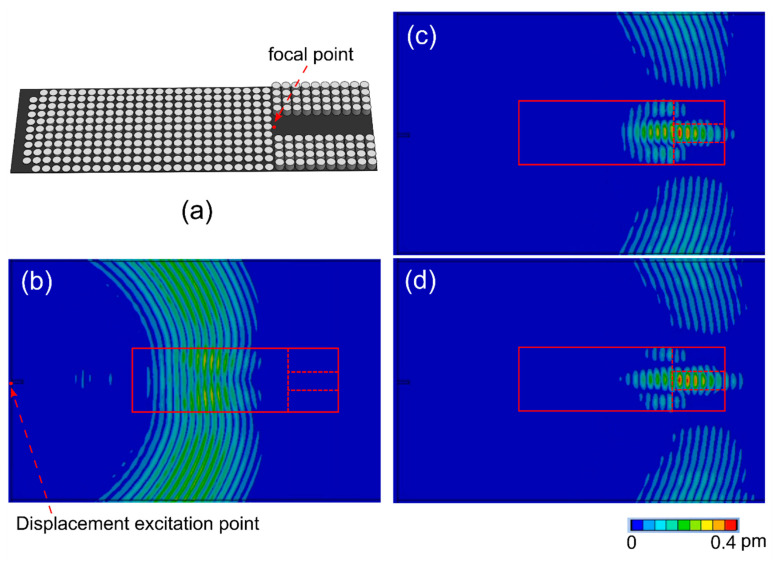
(**a**) Schematic of the GRIN structure with the waveguide channel. Simulated out-of-plane displacement field on the opposite surface of the aluminum plate (**b**) at 208 µs, (**c**) at 283 µs, and (**d**) at 293 µs. Red box shows the location of the GRIN lens and channel, separated by a dashed line.

We next implemented a waveguiding channel to confine the focused Lamb waves after the focal point, as shown in Figure 6a. This confinement structure was similar to the approach for photonic crystals of Chiou et al. [17]. The entrance to the waveguide channel was set to be the location of the focal point based on the LDV measurement results shown in Figure 5. The height of the pillars on each side of the channel was 4 mm, which was higher than three times the height of center pillars of the GRIN structure. This height was chosen such that the bandgap was sufficiently strong above the maximum operating frequency of 50 kHz so that the A_0_ mode could not pass through the lattice. The channel width was 15 mm, i.e., three rows of pillars were removed to allow A_0_ mode propagation through the channel [17].

Numerical simulations were performed to predict the A_0_ mode manipulation by the GRIN lens and channel. The simulations were performed using the transient structural module in ANYSYS. To simulate the PZT actuation, an out-of-plane displacement boundary condition was set at the red point in Figure 6b, with a 5.5 cycle Hanning-windowed burst sine wave at 50 kHz. The aluminum plate and GRIN lens with the waveguide were meshed with a 1 mm square mesh to achieve 12 mesh elements per wavelength. The time step was 1 µs to achieve 20 time steps per wave cycle.

The simulated out-of-plane displacement fields are shown in Figure 6b–d at different simulation time steps. The GRIN structure with the waveguide was located on the opposite side of the plate inside the red box. The red dashed line separates the boundary of the GRIN structure and the channel waveguide. Figure 6b plots the out-of-plane displacement at 208 µs when the A_0_ Lamb wave is propagating through the GRIN structure before reaching the focal point and shows the bending of the wavefront toward the focal point. The wave was focused at the beginning of the waveguide, indicated by Figure 6c. Finally, the wave entered the channel of the waveguide and was confined by the channel, shown in Figure 6d.

**Figure 7:**
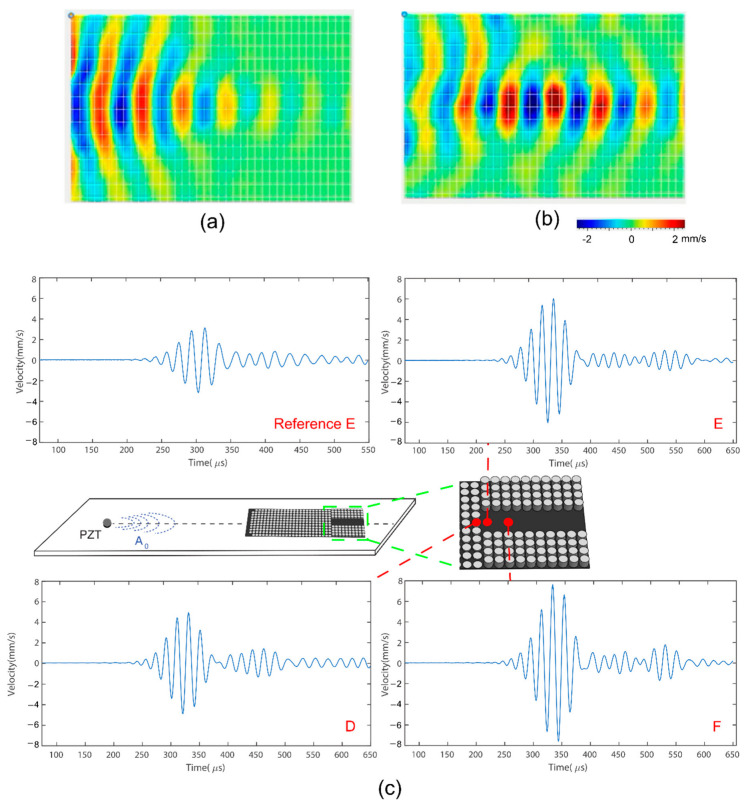
LDV measurements of out-of-plane velocity at (**a**) 298 µs and (**b**) 335 µs in the region surrounding the waveguide channel. Measurements performed on opposite sides of the plate from the GRIN lens. Location of the channel shown as a red box. (**c**) Single point plots of the z-direction velocity with time at different locations before and in the waveguide channel.

We fabricated the GRIN lens with a waveguide channel using the same geometry as in the simulation and experimentally measured its performance. Figure 7a,b plots the LDV measurements of the area around the waveguide channel, indicated by the green dashed line in Figure 7c. The LDV measurements demonstrate that the waveguide effectively confines and guides the A_0_ mode through the channel. Figure 7a shows the A_0_ mode being focused due to the GRIN structure prior to entering the waveguide channel. Subsequently, the A_0_ mode entered the channel and propagated through the waveguide, as shown in Figure 7b, indicating that the wave was trapped within the boundaries of the waveguide channel. Finally, although additional wave packets were observed after the original five cycles, Hanning windowed signal, these were separated from the original signal and so did not distort the signal waveform.

Figure 7c plots single-point measurements in the vicinity of the entrance of the waveguide channel, points D, E, and F. A reference measurement wasalso included for point E, measured without the GRIN structure bonded to the plate. Location D was the last scanning point before the wave entered the channel, and Location E was the first scanning point in the channel. The wave amplitude at Point D increased by 56% compared to the reference point. The wave amplitude was then increased to 92% compared to the reference point at point E. The location of the highest amplitude was point F, at which the amplitude increased by 140% compared to the reference point. Combining the GRIN structure with the waveguide not only confined the wave but also further increased the signal amplitude for a distance in the channel. Therefore, the A_0_ mode amplitude did vary along the channel length, although the gradient was much lower than in the GRIN structure. In the next section, we extract the A_0_ mode at the waveguide channel using an FBG sensor, taking into account the amplitude variation along the channel length.

## 4. Extraction of Lamb Waves with FBG Sensor

**Figure 8:**
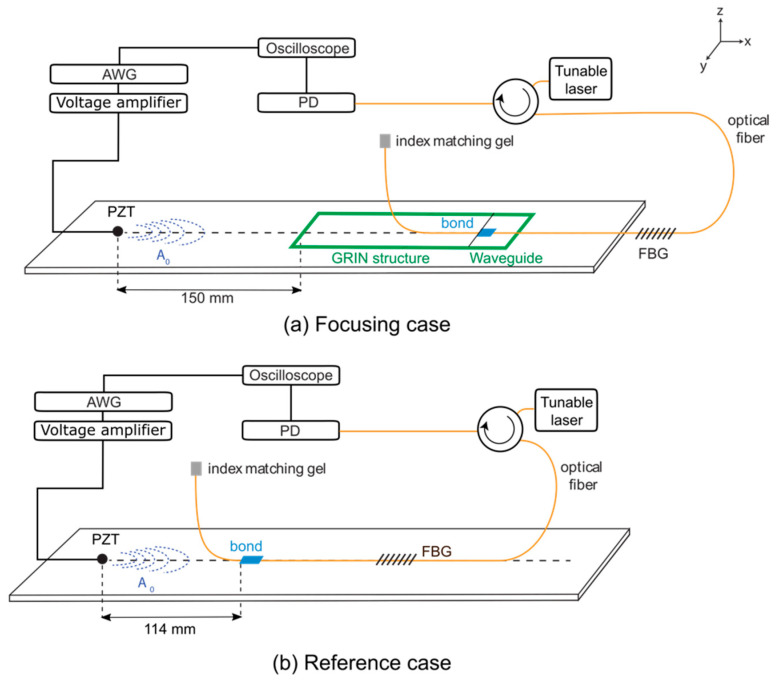
Experimental setup for measuring the amplitude of A_0_ Lamb wave extracted from aluminium plate (**a**) with and (**b**) without amplification by the GRIN structure with waveguide channel.

The response of an FBG sensor, bonded in the waveguide channel region, was measured, varying the location of the sensor along the waveguide channel and the length of the optical fiber bonded in the channel. Figure 8a shows the setup for these experiments. The optical fiber was bonded on the aluminum plate surface, on the opposite side from the GRIN structure, using cyanoacrylate (CA) adhesive (Loctite^®^). The width of the bond area was 10 mm and was centered in the waveguide channel. The FBG was located at 100 mm along the optical fiber from the adhesive bond location. In this remote bonding configuration, the A_0_ Lamb wave propagating through the aluminum plate was coupled with the optical fiber-guided traveling wave through the adhesive bond (blue rectangle) and coupled into a longitudinal (L_01_) wave in the optical fiber [11]. The L_01_ wave propagated along the fiber and was measured with the FBG sensor.

The FBG had a length of 10 mm and was written in a 125 μm diameter SMF-28 optical fiber with a 15 μm thick polyimide coating. The FBG sensor was interrogated based on the edge-filtering method [11]. The narrowband output of the tunable laser (TUNICS PLUS^®^) was initially set to the midpoint of the FBG ascending edge of the reflected spectrum. The variation of axial strain in the optical fiber due to the L_01_ mode induced a wavelength shift in the FBG reflected spectrum, which produced a change in the portion of the light reflected from the FBG and, therefore, a variation in the optical power measured at the photodetector. The change of voltage was converted to strain by first measuring the reflected spectrum by sweeping the tunable laser output and calculating the slope of the spectral edge. The other end of the optical fiber was submersed in an index-matching gel to reduce back reflections.

As the bond location along the channel was varied, and therefore the distance between the FBG sensor and PZT actuator, it was important to have a universal point to reference the measured response to the A_0_ mode amplitude for comparison. This point was chosen at 114 mm (three times the wavelength) from the PZT, as shown in Figure 8b. The dimensions of the adhesive bond for the reference case were 10 mm × 20 mm. The A_0_ mode amplitude along the center line of the waveguide was also independently measured using the LDV to predict the output FBG response and compared to the experimental measurements.

**Figure 9:**
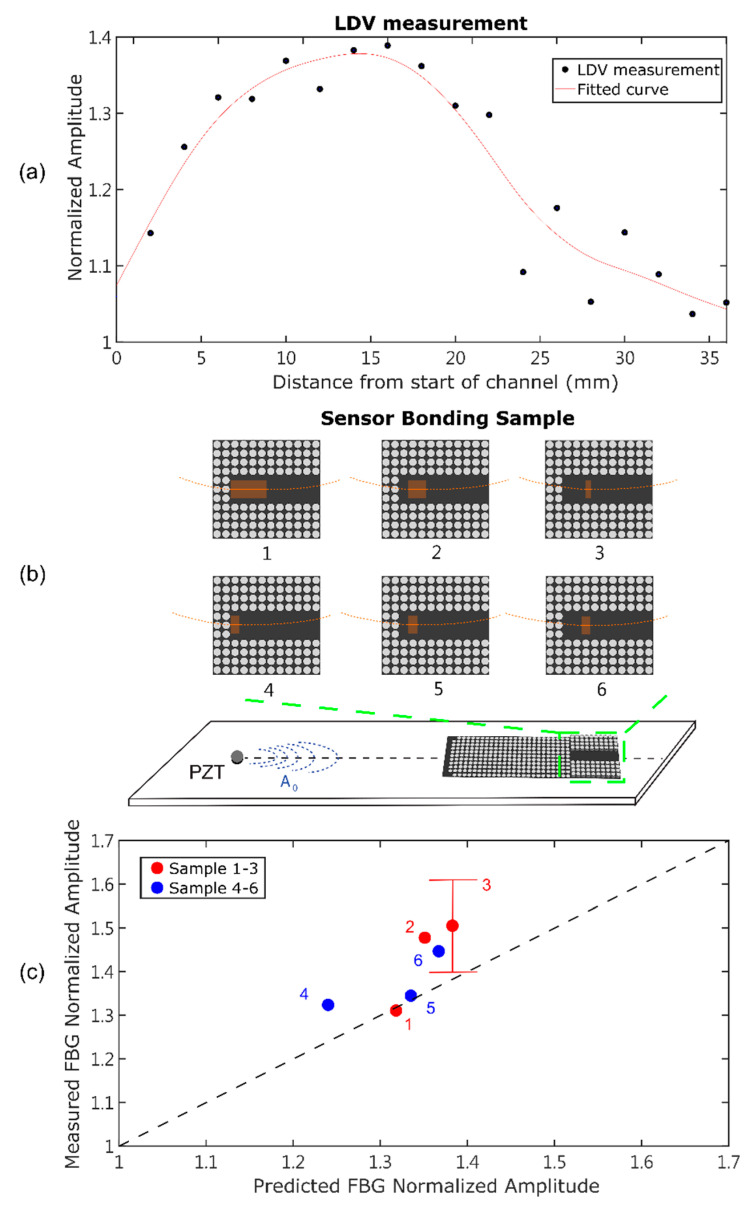
(**a**) Peak-to-peak normalized amplitude of the LDV responses measured along the waveguide channel. Curve fit to data is also plotted. (**b**) Optical fiber bond locations along the waveguide channel for measurements. (**c**) Comparison of measured and predicted FBG response for different bond locations.

Figure 9a plots the peak-to-peak amplitude of the LDV responses measured along the center line of the waveguide channel. The values were normalized to the peak-to-peak amplitude of the reference measurement at point A in Figure 5. There was a large amount of scatter in the measurements due to two factors: the scan points were smaller than the pillar size and therefore were influenced by proximity to the local pillars and the challenge of manually aligning the LDV on the opposite side of the plate from the GRIN lens. Therefore, a polynomial curve was fit to the data, also shown in Figure 9a. Based on the fitted curve, the signal amplitude of the A_0_ mode was expected to be highest at 13 mm into the channel.

Six different FBG bond geometries were experimentally tested, as indicated in Figure 9b and listed in Table 2. The orange regions in Figure 9b are the sensor bonding area. The first three samples (1–3) varied the bond length (from 20 to 2 mm) while including the location of the highest wave amplitude. The second three samples (4–6) kept the bond length constant at 5 mm while varying the bond location along the channel. All samples except sample 3 were rebonded and tested three times. Tests for sample 3 were performed five times because sample 3 had the smallest bond area and therefore was most affected by the manual placement accuracy. The predicted FBG output was also calculated using the average wave amplitude along each bond location from the curve fit to the LDV data in Figure 9a. The measured and predicted FBG output for each bond case are plotted against each other in Figure 9c. The data were normalized to the reference case.

Sample 1 had the longest bond length, and the measured and predicted FBG sensor outputs matched closely. This bond configuration was also the least sensitive to the manual placement of the bond because of the long bond length. Samples 2 and 3 had shorter bond lengths, and the measured FBG sensor output was higher than the predicted value. The sensitivity of an FBG sensor to an input wave varied with the ratio of the wavelength to the bond length (λ/L) [21]. The sensitivity of the FBG to the wave increased with decreasing bond length (for a constant input wave frequency and windowing function) up to a critical value. The increase in the measured vs. the predicted signal output may have been due to this effect, although a significant increase difference was not observed between Samples 2 and 3. The sensitivity as a function of (λ/L) was not included in the calculations plotted in Figure 9c because it was influenced by the exact waveform and distribution along the bond. Further studies are required to capture the waveform with a resolution finer than Figure 9a for these calculations.

Sample 3 had the shortest bond length, only 2 mm, and showed much variability in the measured response. The error bars for this case are plotted in Figure 9c. The accuracy of the manual placement was greater than 1 mm, and the waveform amplitude was rapidly varying in this region; therefore, the actual wave amplitude along the bond length varied significantly between the different tests. The error bars for the other cases were negligible and therefore were not plotted.

Samples 4, 5, and 6 had the same bond length; therefore, we expected to see an increasing signal from samples 4 to 5 to 6 due to the average wave amplitude along the bond. The measured signal output from samples 4 and 5 was approximately the same, but then the signal output from sample 6 was higher. The bond case with the largest difference between the predicted and measured output was Sample 4, which was located at the beginning of the channel. This was most likely due to the fact that the confinement of the wave in the channel was increasing over this length; therefore, the location of the FBG placement was critical. Importantly, the signal amplification was equal to or higher than that predicted for all samples, with a maximum amplification of approximately 1.5 achieved. This amplification was achieved without optimization of the GRIN lens or the waveguide channel.

These FBG measurements show that the A_0_ mode Lamb wave was successfully amplified, collimated, and extracted through the GRIN structure with the waveguide channel.

## 5. Conclusions

This work demonstrates that the GRIN structure focuses the A_0_ Lamb wave in the plate and the waveguide channel prevents expansion of the wavefront after the focal point. The waveform was successfully extracted using a surface-bonded FBG in the channel. While the focused wave energy can be confined using the waveguide channel design, the FBG sensor location for signal extraction should be carefully selected to optimize the extraction of signal energy. In the future, the waveguide configuration could also be optimized for the geometry and directionality of other sensors. The cost-effectiveness of producing a GRIN structure using 3D printing techniques and mounting using commercially available adhesives could be beneficial for practical SHM applications using ultrasound inspection techniques.

## Figures and Tables

**Table 1:** Pillar heights for phononic crystal GRINS lens. Values are in mm.

	Row #										
Column	1	2	3	4	5	6	7	8	9	10	11
1	0	0	0	0	0	0	0	0	0	0	0
2	0	0.25	0.33	0.37	0.39	0.40	0.39	0.37	0.33	0.25	0
3	0.10	0.50	0.66	0.74	0.78	0.80	0.78	0.74	0.66	0.50	0.10
4–35	0.15	0.75	0.99	1.11	1.17	1.20	1.17	1.11	0.99	0.75	0.15

**Table 2:** FBG bonding parameters.

Sample #	Length, a (mm)	Start of Bond (mm)	End of Bond (mm)
1	20	0	20
2	10	5	15
3	2	13	15
4	5	0	5
5	5	5	10
6	5	10	15

## Data Availability

The data presented in this study are available on request from the corresponding author.
